# Supplementary material for: Chromothripsis during telomere crisis is independent of NHEJ, and consistent with a replicative origin
Source: Genome Res. 2019 May;29(5):737–49. doi: 10.1101/gr.240705.118 (PMC6499312; doi:10.1101/gr.240705.118)
Supplement: Supplemental Material [file supp_gr.240705.118_Supplemental_file_1.zip › contigs/annotated_contigs/DB77/contig.2.DB77_length_478_mean_cov_27.7677824268.docx]

**DB77_length_478_mean_cov_27.7677824268**

```
CTCCTGCTAAATAAATGGGGGCAGTAGATTTGCATCGGTT|TCCACATTACATTACTCATAACTGTGGCCTTCCTAATTTCTCATGTCC
                                         >chrUn_gl000219:56396-56763 - E=8e-115
CTAAGCACTTATTACAATGTCTCAGCAGCTTTCTCTCAGCCTCTCAAAATATTAGTCATCATGAAAATAAATGTCTGATGTTAAAGCAT

CAGGGGTCAGTTGTATTGTGACACCTAAAATTTCATGTAGCACCCCACCATGTCCTCAGCCTAATGACTTTCATGTCCCCACCAGACTG

CAAAACTCTGAATTAGTCAAGCACATCCCTGAAGGGACCAGGGGTTACCCCACCCTCTGATTTCTACCAAGCCTGCCTCCCACACCCTC

TGTTCCAGAGTGCAACCCTGGGTAGACCTGCATAAATGCAGTGTCATCCCC|CTGGCTGAGTATGTGTGACGAG|TAAATTACTGGGAA
                                                                          >chr9:6842585
TTTCGTCTGCTCAGTGTTTTGTCTGCAGCCATTCCC6-68425910 - E=2e-12
```
